# Laser doppler flowmetry as a diagnostic tool to detect gingival inflammation: a systematic review

**DOI:** 10.1186/s12903-025-06608-9

**Published:** 2025-07-31

**Authors:** Marie Sophie Katz, Mark Ooms, Marius Heitzer, Philipp Winnand, Anna Bock, Maurice Klein, Frank Hölzle, Ali Modabber

**Affiliations:** Department of Oral and Maxillofacial Surgery, University Hospital RWTH Aachen, Pauwelsstraße 30, Aachen, Germany

**Keywords:** Laser-Doppler flowmetry, Perfusion, Gingivitis, Periodontitis, Inflammation

## Abstract

**Background:**

The aim of this systematic review was to evaluate whether laser Doppler flowmetry (LDF) as a diagnostic tool is effective in detecting gingival inflammation.

**Methods:**

This review was conducted and reported according to the Preferred Reporting Items for Systematic Reviews and Meta-Analyses (PRISMA) and registered at the International Prospective Register of Systematic Reviews (PROSPERO) (registration number CRD42025633576). Two authors independently performed searches in PubMed, Embase, the Cochrane Library, and Scopus.

**Results:**

In total, 1317 studies were identified, of which 10 met the inclusion criteria after full-text screening. All studies compared perfusion with a healthy control group, seven included a gingivitis group, five included patients with periodontitis, and none included patients with peri-implant disease. Higher blood flow values were found in patients with chronic gingivitis compared to healthy controls, while patients with experimental gingivitis showed no significant difference. Blood flow values in patients with periodontitis showed controversial results.

**Conclusions:**

LDF shows potential utility in detecting chronic gingival inflammation and altered perfusion patterns between healthy individuals and those with gingival or periodontal inflammation. However, reliably grading perfusion across disease stages and distinguishing between acute gingivitis and chronic periodontitis remains challenging. Notably, there is a lack of data regarding the use of LDF in peri-implant tissues, representing a significant gap in current research. At present, established clinical parameters such as probing depth and bleeding on probing (BOP), remain the gold standard in routine periodontal diagnostics.

**Supplementary Information:**

The online version contains supplementary material available at 10.1186/s12903-025-06608-9.

## Background

As hyperperfusion is one of the main features of gingival inflammation, bleeding on probing (BOP) is the key parameter in the detection of gingivitis and periodontitis, as well as in the diagnosis of peri-implant disease [1–3]. Indices to grade the extent of bleeding and inflammation include the Gingival Bleeding Index by Ainamo and Bay [4], the Gingival Index (GI) by Löe and Silness [5, 6], and or the Papilla Bleeding Index (PBI) by Saxer and Mühlemann [7]. However, it has been shown that BOP can strongly correlate with pressure applied on the periodontal probe and be influenced by smoking habits [8–13]. Hence, the results can be interpreted as false positives or false negatives.

On a microvascular level, the shift from a healthy to an inflamed gingiva correlates with changes in gingival perfusion, and periodontitis is associated with the proliferation of small blood vessels and the enhanced vascularization of periodontal ligaments [14, 15]. Early detection of gingival inflammation is crucial to prevent further attachment loss, maintain periodontal health, and secure implant survival [16].

Various imaging modalities have been explored to assess periodontal and peri-implant perfusion, including ultrasonic devices, laser speckle contrast imaging, reflection photoplethysmography, and laser Doppler flowmetry (LDF) [17–20]. Among these, LDF stands out as a frequently used technique—not only in dental research but also in broader medical contexts, such as monitoring microvascular circulation in skin transplants and detecting perfusion deficits in conditions like diabetes [21–25].

LDF is based on the detection of changes in the wavelength that occur as moving red blood cells reflect light (Doppler effect) [26]. It enables continuous measurement of blood flow and provides real-time data on velocity in arbitrary units (AU). Variations in blood flow values may indicate increased perfusion or tissue ischemia compared to other sites or to previous measurements [27–30]. However, the method is sensitive to several limitations, including variability due to probe angulation, unstable positioning, and movement artifacts. These factors can affect measurement reliability and inter-study comparability [27, 31].

Intraoral applications of LDF have been used to assess wound healing and vascularization following periodontal procedures [32, 33], evaluate smoking-related gingival vasoconstriction [34–36], analyze the effects of various substances, such as nitric oxide on perfusion, and detect gingival inflammation [17, 28, 37–39].

Advances in probe design, including smaller devices and custom splints for probe stabilization, have aimed to improve consistency and usability in clinical settings [31, 40].

Although multiple dental research studies have employed this technology, a systematic overview evaluating its effectiveness in detecting gingival and periodontal inflammation is still lacking. The aim of this systematic review was to assess whether LDF is an effective method for diagnosing gingival inflammation to determine whether a correlation exists between perfusion values and clinical indices.

## Methods

### Protocol development and eligibility criteria

 This review was conducted and reported according to the Preferred Reporting Items for Systematic Reviews and Meta-Analyses (PRISMA) [41]. The protocol was registered at the International Prospective Register of Systematic Reviews (PROSPERO) with registration number CRD42025633576 [42].

Prior to initiation, a protocol including all aspects of a systematic review methodology was developed. This included the definition of a focused question, a PICOS (patient, intervention, comparison, outcome, and study design) question, a defined search strategy, study inclusion criteria, determination of outcome measures, screening methods, and data extraction, analysis, and synthesis.

#### Defining the focused question

The following focused question was defined: “Is laser Doppler flowmetry a useful diagnostic tool to detect gingival inflammation around natural teeth or implants?”.

#### PICOS question

P: Patients with natural teeth or implants.
I: Undergoing perfusion measurements with laser Doppler flowmetry.
C: Compared to clinical parameters.
O: Correlation in blood flow values.
S: Clinical studies in humans (Cross-sectional studies and prospective cohort studies).

#### Search strategy

Two authors (MSK and AM) independently performed an electronic search in PubMed, Embase, the Cochrane Library, and Scopus on September 29, 2024. Articles published up to September 1, 2024, were considered. No time restrictions were applied in the search. Although no language limits were set during the search process, only articles written in German or English were considered for inclusion.

#### Search terms

The database search strategy used the following combinations of key words: (“laser Doppler flowmetry” OR “laser Doppler Flowmetry” OR “laser Doppler”) AND (“gingival” OR “gingiva” AND “perfusion” OR “microcirculation” AND “inflammation” OR “gingivitis” OR “peri-implant mucositis” OR “peri-implantitis” OR “periodontitis”). The full search strings as applied in each database are provided in Supplementary Table 1. Additionally, the reference lists of the articles included in the present review were screened.

#### Study selection and inclusion criteria

The study selection criteria were studies in German or English. Only clinical studies (Cross-sectional studies and prospective cohort studies) in patients with gingival or periodontal inflammation around teeth and dental implants measured by LDF were included. The review included studies on generally healthy, non-smoking individuals, as well as studies involving smokers and patients with diabetes. Studies using other measurement techniques, such as laser speckle or other ultrasonic diagnostic methods, were excluded, as were studies investigating the use of LDF for monitoring wound healing after surgical procedures, as well as retrospective studies or case reports.

#### Screening and selection of studies

Two independent reviewers (MSK and AM) screened the titles and abstracts of the selected studies based on the question, “Is laser Doppler flowmetry a useful diagnostic tool to detect gingival inflammation around natural teeth or implants?” Discrepancies were solved by discussion between the two reviewers (MSK and AM) and a judge (MO). Cohen’s Kappa coefficient was calculated to measure the agreement between the two reviewers. The full-text articles were obtained if the answer to the screening was “yes” or “uncertain.”

#### Data extraction and analysis

The following data were extracted: author(s), year of publication, type of study, number of patients, type of gingival or periodontal disease, measurement procedure, primary outcome measurement, and significance value. All studies were classified according to the study design to provide an overview of all studies matching the search criteria and those excluded. Afterwards, the outcomes were compared in separate tables and discussed.

##### Quality assessment and risk of bias (RoB)

The risk of bias was assessed using the Risk Of Bias In Non-randomised Studies – of Interventions (ROBINS-I) tool [43]. This tool evaluates the quality of non-randomized studies across seven domains:

1. Bias due to confounding
2. Bias in the selection of participants
3. Bias in the classification of interventions
4. Bias due to deviations from intended interventions
5. Bias due to missing data
6. Bias in the measurement of outcomes
7. Bias in the selection of the reported result

Each domain is rated as having a low, moderate, serious, or critical risk of bias, or as providing no information.

## Results

### Selection of studies

**Fig. 1:**
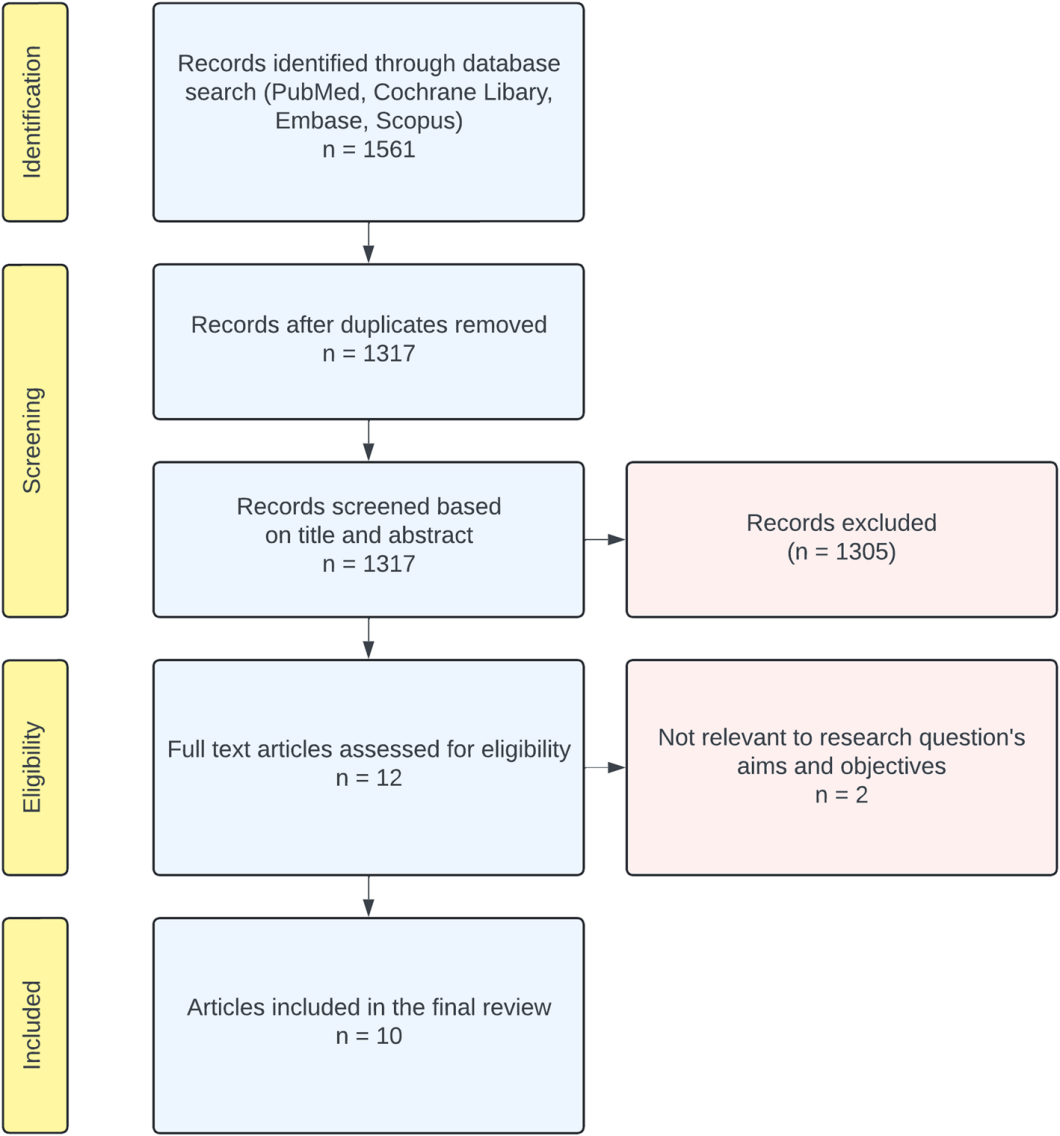
Flow diagram of study identification, screening, and inclusion process adapted from PRISMA

The database searches identified 1561 articles in PubMed, Embase, the Cochrane Library, and Scopus. After removing duplicates, 1317 remained, and the two independent researchers screened the titles and abstracts. Subsequently, 12 articles were found to meet the inclusion criteria and were selected for full-text analysis (inter-reviewer agreement κ = 0.450) (Fig. 1). One study by Develioglu et al. [39] that reported blood flow differences between the gingiva adjacent to fixed restorations compared with the gingiva next to natural teeth was excluded because it did not contain an analysis of healthy patients vs. patients with gingivitis. Another study by Develioglu et al. that evaluated the correlation between LDF measurements and clinical parameters was omitted, because it excluded patients with gingivitis and periodontitis [44]. Finally, 10 studies were considered relevant and were included in this systematic review.

### Study characteristics

Classifying the types of studies included, six clinical cross-sectional studies analyzed patients at a single measurement date [28, 45–49], and four studies were clinical cohort studies [27, 38, 50, 51], of which two induced experimental gingivitis [27, 50], and two evaluated perfusion before and after periodontal treatment [38, 51]. One of the clinical cross-sectional studies evaluated microcirculatory parameters in gingival tissues before and after tooth extraction; for the purpose of this review, only the preoperative measurements were included [49].

All studies compared perfusion to a healthy control group, seven studies included a group of patients with gingivitis [27, 28, 38, 46, 47, 50, 51], five studies included patients with periodontitis [45–49] (Table 1). None of the reviewed studies included patients with peri-implant disease.

**Table 1:** Study population characteristics

Authors (year)	Type	Number of patients	Type of gingival or periodontal disease	Groups
Baab et al. (1990) [45]	Clinical cross-sectional study	20 patients (aged 17–35 years)	10 patients with healthy gingiva 10 patients with localized juvenile periodontitis (3) or rapidly progressive periodontitis (7)	Healthy control vs. periodontitis
Matheny et al. (1993) [50]	Prospective clinical cohort study	10 patients (all male, aged 18–30 years)	10 patients with healthy gingiva, measured once in healthy state and after induction of experimental gingivitis	Healthy control vs. gingivitis
Kerdvongbundit et al. (2002) [46]	Clinical cross-sectional study	60 patients (all female, aged 20–35 years)	20 patients with healthy gingiva 20 patients with moderate gingivitis 20 patients with periodontitis	Healthy control vs. gingivitis vs. periodontitis
Kerdvongbundit et al. (2003) [51]	Prospective clinical cohort study	24 patients (all female, aged 18–25 years)	12 patients with healthy gingiva 12 patients with moderate gingivitis	Healthy control vs. gingivitis
Gleissner et al. (2006) [27]	Prospective clinical cohort study	21 patients (13 females, 8 males; aged 23–63 years)	10 patients with healthy gingiva, measured once in healthy state and after induction of experimental gingivitis 11 patients with chronic gingivitis	Healthy control vs. experimental gingivitis vs. chronic gingivitis
Rodríguez-Martínez et al. (2006) [47]	Clinical cross-sectional study	60 patients (38 females, 22 males; aged 30–60 years)	9 patients with healthy gingiva (no gingivitis, no periodontitis) 30 patients with isolated gingivitis (no periodontitis) 3 patients with isolated periodontitis (no gingivitis) 18 patients with gingivitis and periodontitis	Healthy control vs. isolated gingivitis vs. isolated periodontitis vs. gingivitis combined with periodontitis
Develioglu et al. (2014) [48]	Clinical cross-sectional study	48 patients (no information about sex; Group 1 aged 65.31 ± 5.98 years; Group 2 aged 51.62 ± 9.56 years; Group 3 aged 23.06 ± 4.44 years)	16 patients with chronic periodontitis (Group 1) 16 patients with chronic periodontitis and diabetes (Group 2) 16 healthy patients (Group 3, control)	Healthy control vs. chronic periodontitis vs. chronic periodontitis with diabetes
Canjãu et al. (2015) [38]	Prospective clinical cohort study	10 patients (7 females, 3 males; aged 20–30 years)	10 patients with healthy gingiva and sites with gingivitis (at least one of each), measured once and after periodontal treatment	Healthy control vs. gingivitis
Katz et al. (2023) [28]	Clinical cross-sectional study	114 patients (61 females, 53 males; mean age 32.6 years)	50 patients with gingivitis and 64 patients without gingivitis	Healthy control vs. Gingivitis
Mayr et al. (2024) [49]	Clinical cross-sectional study	37 patients (17 females, 20 males; mean age 53 years)	7 periodontally healthy patients, non-smokers 10 anamnestically healthy patients with periodontitis, non-smokers 10 anamnestically healthy patients with periodontitis, smokers 10 patients with diabetes and periodontitis, non-smokers	Healthy control vs. periodontitis

Most studies measured perfusion only around anterior teeth [27, 38, 46–48, 50, 51]. One study measured at the left upper first molar [45], one study evaluated perfusion on the papillae of both anterior and posterior teeth [28], and one study did not include information about the region measured [49] (Table 2). All measurements took place at the buccal site of dentition. Some studies analyzed perfusion at the marginal gingiva [45, 47, 50], one analyzed it at the papillae [28], one analyzed it in the area of the mucogingival junction [49], and other studies used several points next to the teeth [27, 38, 46, 48, 51]. Kerdvongbundit et al. reported significant differences in perfusion values across various measurement sites [46]. The LDF probes used in the studies were made by four different manufacturers, and most studies used a splint [27, 38, 46–48, 50, 51] or an orthodontic ligature [45] to stabilize the probe during the measurements. Gleissner et al. showed that hand-held and splint-stabilized measurements differed significantly [27]. Six studies found higher blood flow values in the patients with gingivitis compared to the healthy controls [27, 28, 38, 46, 47, 51]. In the study by Gleissner et al., however, a significant difference was observed only in patients with chronic gingivitis; the difference between experimental gingivitis and healthy gingiva was not statistically significant [27]. The studies by Kerdvongbundit et al. and Canjãu et al. showed that blood flow values normalized to a lower level after gingivitis treatment, although this occurred over a period of two weeks to three months [38, 51]. Conversely, Matheny et al. found significantly decreased blood flow in patients with gingivitis, but a significant increase in the number of visible vessels [50].

**Table 2:** Comparison of LDF measurement procedures, clinical parameters, and outcomes of the included studies

Author (year)	Measurement procedure	Clinical parameters and exclusion criteria	Main outcome	*p*-value
Baab et al. (1990) [45]	Measurement on buccal site of left upper first molar (tooth 26), stabilized by an elastic orthodontic ligature Resting values were measured, and afterwards, the gingiva was cooled to 19 °C by a stream of cold air, followed by a 12 min rewarming period. This procedure was repeated twice LDF^a^ manufacturer: Periflux PF-2, Perimed KB, Stockholm, Sweden No information about probe dimensions	• Clinical diagnosis of juvenile periodontitis or rapidly progressive periodontitis based on clinical and radiologic features and a microbiological culture • *Exclusion criteria* - Smoking 3 h prior - Drinking caffeine-containing beverages 3h prior	Gingival blood flow in young patients with periodontitis recovered more rapidly after cooling than in healthy controls No information about differences in baseline values between healthy controls and patients with periodontitis	Patients with periodontitis showed significantly faster recovery of blood flow after cooling: *p* < 0.03 during first procedure, *p* < 0.001 during second procedure
Matheny et al. (1993) [50]	LDF measurements on the facial marginal gingiva on teeth 7, 8, and 9 (= upper right lateral incisor, upper right medial incisor, and upper left medical incisor), stabilized by an acrylic probe holder. Combined with videomicroscopic measurements to picture the number of vessels LDF manufacturer: TSI (St. Paul, Minnesota, USA) No information about probe dimensions	• Plaque index • GI^b^ • Gingival Bleeding Index • Gingival crevicular fluid volume • Pocket depth • Systolic, diastolic, and mean arterial blood pressure, peripheral oxygen saturation, and tidal carbon dioxide concentration • *Exclusion criteria* - Smokers - Fewer than 20 natural teeth - Fewer than 5 continuous upper anterior teeth - Anterior restorations - Systemic diseases - Intake of drugs that could alter microcirculation - Clinical gingivitis or periodontitis before experimental induction (mean GI score > 1.0 or probing depth > 4 mm) - Carious lesions, other oral pathology, orthodontic bands, removable protheses - Use of antibacterial or anti-inflammatory substances within 1 month of screening	No change in superficial capillary blood velocity but significant decrease in gingival regional blood flow after induction of gingivitis; significant increase in number of visible vessels	No *p*-values available
Kerdvongbundit et al. (2002) [46]	Six maxillary anterior teeth: LDF measurements on facial free gingiva, interdental gingiva, attached gingiva and alveolar mucosa. Acrylic stent used to stabilize the probe LDF manufacturer: Moor Instruments (Axminster, England) No information about probe dimensions	• Qualitative plaque index • GI • Gingival Bleeding Index • Tooth mobility • Pocket depth • Clinical attachment level • *Exclusion criteria* - Smokers - Current orthodontic/prosthetic treatment, injuries, oral pathology - Intake of anti-inflammatory/antibacterial substances	Healthy gingiva showed significantly lower blood flow values compared to gingiva affected by gingivitis or periodontitis. Disease progression could not be shown with LDF, as blood flow values were not significantly different between gingivitis and periodontitis Blood flow values differ significantly, depending on where measurement is taken	*p* < 0.001 between healthy sites compared to sites with gingivitis and sites with periodontitis at all measurement points No significant difference in blood flow values between sites with gingivitis and periodontitis (no *p*-value reported) *p* < 0.001 at different measurement points within same group
Kerdvongbundit et al. (2003) [51]	Six maxillary anterior teeth: LDF measurements on facial free, interdental, and attached gingiva and alveolar mucosa. Acrylic stent used to stabilize probe at 90° angle to gingival surface. Patients underwent periodontal treatment, including oral hygiene instructions and scaling; follow-up after 1 and 3 months LDF manufacturer: Moor Instruments (Axminster, England) No information about the probe dimensions	• Plaque index • GI • Gingival Bleeding Index • Tooth mobility • *Exclusion criteria* - Smokers - Current orthodontic/prosthetic treatment, injuries, oral pathology - Intake of anti-inflammatory/antibacterial substances	Moderate gingivitis changed to mild gingivitis after 1 month and healthy after 3 months. Blood flow values significantly lower in healthy group compared to gingivitis group before treatment. After treatment, blood flow values restored to same level as healthy gingiva. Gingival morphology of inflamed sites exhibited irregular margins at baseline, which normalized to a physiological micromorphology after treatment	*p* < 0.001 for blood flow values of gingivitis vs. healthy sites before periodontal treatment After treatment, no significant difference remained between the two groups (*p* > 0.01) *p* < 0.001 at different measurement points within same group
Gleissner et al. (2006) [27]	LDF measurements on facial marginal gingiva on 13 gingival sites of teeth 6–11 (= left upper canine to right upper canine; papillae and marginal gingiva), measured twice stabilized by acrylic probe holder and once measured handheld. Measured baseline at healthy sites and sites affected by chronic gingivitis and after 8–20 days after installing of experimental gingivitis LDF manufacturer: TSI (St. Paul, Minnesota, USA) Probe dimensions: 0.8 mm needle-shaped	• Plaque index • GI • Probing depth and clinical attachment loss • *Exclusion criteria* - Smokers - Pregnancy - General diseases - Long-term medication (except contraceptives) - Intake of anti-inflammatory/antibacterial medication within 1 month before screening - Orthodontic bands or fillings on test teeth - Probing depths > 2 mm or clinical attachment loss > 1 mm	LDF values differed significantly between splint stabilized vs. handheld. LDF values increased in experimental gingivitis, but difference not significantly different from healthy gingiva blood flow. Blood flow in chronic gingivitis significantly higher than in experimental gingivitis and healthy gingiva	*p* < 0.05 for splint stabilized vs. handheld *p* < 0.05 for blood values of chronic gingivitis compared to healthy gingiva *p* < 0.05 for blood values of chronic gingivitis compared to experimental gingivitis *p* < 0.05 for blood values grouped by GI (except for GI = 0 and GI = 1)
Rodríguez-Martínez et al (2006) [47]	LDF measurements on labial marginal gingiva on lower left lateral incisor. Twice measured stabilized by horseshoe-shaped tray LDF manufacturer: PF5001 main unit and PF5010 LDPM unit, Perimed, Stockholm, Sweden No information about probe dimensions	• GI • Probing depth and clinical attachment loss • *Exclusion criteria* - Smokers - Pregnancy - Evident genetic diseases - Epilepsy - Persons with renal transplantation - Use of drugs that affect blood glucose, other than hypoglycemic drugs	Gingival perfusion significantly higher in patients with isolated gingivitis compared to healthy patients and significantly lower in patients with periodontitis than in healthy patients. Patients with periodontitis and gingivitis combined had significantly lower blood flow values than healthy patients and patients with only gingivitis GI influenced the gingival perfusion index (positive prediction) No significant difference between patients with and without diabetes	*p* < 0.005 for gingival perfusion in patients with gingivitis *p* < 0.001 for gingival perfusion in patients with periodontitis
Develioglu et al. (2014) [48]	LDF measurements on facial marginal gingiva of the six upper anterior teeth (= left upper canine to right upper canine; papillae and marginal gingiva), stabilized by an acrylic splint LDF manufacturer: Periflux 4001 Master LDF device, Perimed, Stockholm, Sweden Probe dimensions: diameter 1 mm, wavelength 780 nm	• GI • Probing depth and clinical attachment loss • Plaque index • *Exclusion criteria* - Smokers - Pregnancy - General diseases - Long-term medication and antibacterial or anti-inflammatory medication within last month	LDF values significantly different between the three groups Blood flow values significantly lower in healthy group compared to group with chronic periodontitis (*p* < 0.005) and group with chronic periodontitis and diabetes (*p* < 0.005). Patients with chronic periodontitis showed lower blood flow values than patients with periodontitis and diabetes (*p* < 0.005) Positive correlation between GI and blood flow values (*p* < 0.005)	*p* < 0.005 for blood flow between healthy and chronic periodontitis *p* < 0.005) for blood flow between healthy and chronic periodontitis with diabetes *p* < 0.005 for blood flow between periodontitis with and without diabetes
Canjãu et al (2015) [38]	LDF measurements on facial marginal gingiva on 20 gingival sites of teeth 6–11 (= left upper canine to right upper canine; papillae and marginal gingiva), stabilized by silicon holder. Measured baseline at healthy sites and sites affected by gingivitis and 24 h, 7 days and 14 days after periodontal treatment LDF manufacturer: VMS-LDF2 probe VP3 10 mm S/N 2482; MoorLab Instruments (Axminster, England) Probe dimensions: diameter 1.5 mm	• Plaque deposits • Changes in gingival color or texture • BOP^c^ • *Exclusion criteria* - Smokers - Pregnancy - Systemic diseases - Long-term medication (except contraceptives) - Intake of anti-inflammatory/antibacterial medication within 1 month before screening - Use of antibiotics within past 6 months - Orthodontic bands or fillings on test teeth - Probing depths > 2 mm or clinical attachment loss > 1 mm	LDF values significantly higher in sites with gingivitis compared to sites without gingivitis at baseline. 24 h after treatment, increased values compared to the baseline values. 7 days after treatment, blood flow not restored to baseline values in both groups. 14 days after treatment, no significant differences remained between the groups. No information about correlation of BOP, plaque and gingival appearance, and blood flow values	*p* < 0.002 for baseline values between sites with and without gingivitis. *p* = 0.645 between the groups 14 days after periodontal treatment
Katz et al (2023) [28]	LDF measurements taken handheld on facial papillae on 22 sites beginning from upper right first molar to upper left first molar, then lower left first molar to lower right first molar. Measured before clinical examination to avoid influence of mechanical intervention on perfusion measurements LDF manufacturer: LSX-41 gingival probe, “oxygen to see” (O2C) device, LEA-Medizintechnik, Gießen, Germany Probe dimensions: 5 × 2 mm; measurement depth of 1 mm	• BOP and PBI^d^ • Plaque index • Pocket depth • Biotype • *Exclusion criteria* - Patients who smoked less than 2 h before examination - Signs of acute infection or intraoral swelling - Patients with missing teeth or dental implants - Patients with diabetes or other diseases that affect peripheral vascular perfusion - ASA^e^ class ≥ 3	Mean oxygen saturation SO_2_ (%), mean relative amount of hemoglobin rHb (AU), and mean blood flow (AU) all differed significantly between the groups with and without gingivitis (*p* = 0.005, *p* < 0.001, and *p* < 0.001, respectively). Smoking observed significantly more often in gingivitis group (*p* = 0.007) but no significant impact on perfusion parameters measured (gingivitis vs. non-gingivitis patients: mean SO_2_ *p* = 0.207, mean rHb *p* = 0.724, and mean flow *p* = 0.171)	*p* < 0.001 for mean blood flow between patients with and without gingivitis. Optimal cutoff value of mean blood flow for predicting gingivitis determined to be > 40 AU. *p* < 0.001 for mean blood flow values compared between sites with and without BOP. *p* < 0.001 for mean probing depths ≥ 2 mm compared to probing depths < 2 mm. *p* = 0.171 for mean blood flow between smokers and non-smokers
Mayr et al. (2024) [49]	LDF measurements taken handheld on vestibular side in area of mucogingival junction and alveolar mucosa of the tooth that was extracted afterwards and on the vestibular mucosa of the contralateral tooth (= control tooth). No information about locations measured Applied with a continuous contact pressure of 0.25 N, three individual measurements per tooth LDF manufacturer: LF-2 gingival probe, “oxygen to see” (O2C) device, LEA-Medizintechnik, Gießen, Germany No information about probe dimensions	• No information on how the periodontitis diagnosis was archived clinically • *Exclusion criteria* - Diseases relevant to patient’s medical history requiring medication that altered coagulation or blood flow - Severe obesity - Antihypertensive therapy or hypertonia	Mean blood flow statistically lower in healthy patients compared to patients with periodontitis (*p* = 0.065). Mean oxygen saturation SO_2_ (%) significantly higher in healthy patients than in periodontitis patients (*p* = 0.038)	*p* = 0.065 for blood flow values between healthy vs. periodontitis patients *p* = 0.012 for anamnestically healthy patients with and without periodontitis (non-smokers, no diabetes) *p* = 0.109 for healthy vs. periodontitis and smoking *p* = 0.191 for periodontitis patients with vs. without diabetes

^a^*LDF* laser Doppler flowmeter, ^b^*GI* Gingival Index, ^c^*BOP* bleeding on probing, ^d^*PBI* Papilla Bleeding Index (PBI), ^e^*ASA* American Society of Anesthesiologists

Baab et al. described faster recovery of gingival blood flow in young patients with periodontitis after cooling than in healthy controls, but their study did not contain any information about differences in baseline values between the healthy controls and the periodontitis patients [45].

Rodríguez-Martínez et al. also compared healthy patients to patients with periodontitis, and found that gingival perfusion was significantly lower in the patients with periodontitis [47]. They also showed that the patients who had a combination of periodontitis and gingivitis had significantly lower blood flow values than the healthy patients and the patients who had only isolated gingivitis [47]. In contrast, Mayr et al. and Develioglu et al. found higher blood flow values in patients with chronic periodontitis than in healthy controls [48, 49]. Kerdvongbundit et al. also reported significantly higher blood flow values in patients with periodontitis compared to healthy controls, but they did not find any difference in flow between the patients with gingivitis and those with periodontitis [46]. However, while Mayr et al. and Rodríguez-Martínez et al. found that gingival perfusion was similar between the medically healthy patients and patients with diabetes [47, 49], Develioglu et al. measured higher blood flow values in patients with chronic periodontitis and type II diabetes [48, 49].

Smokers were excluded in most studies [27, 38, 46–48, 50, 51]. Two studies included smokers, provided they had not smoked immediately prior to the examination [28, 45], and Mayr et al. also included active smokers [49].

Katz et al. found no difference between non-smokers and smokers who had not smoked for two hours prior to the examination [28]. Mayr et al. reported a statistically significant difference between healthy individuals and non-smoking periodontitis patients, but not between healthy individuals and smoking periodontitis patients [49].

The study by Baab et al. did not report values comparing regular smokers and non-smokers [45].

### Risk of bias in the individual studies

**Fig. 2:**
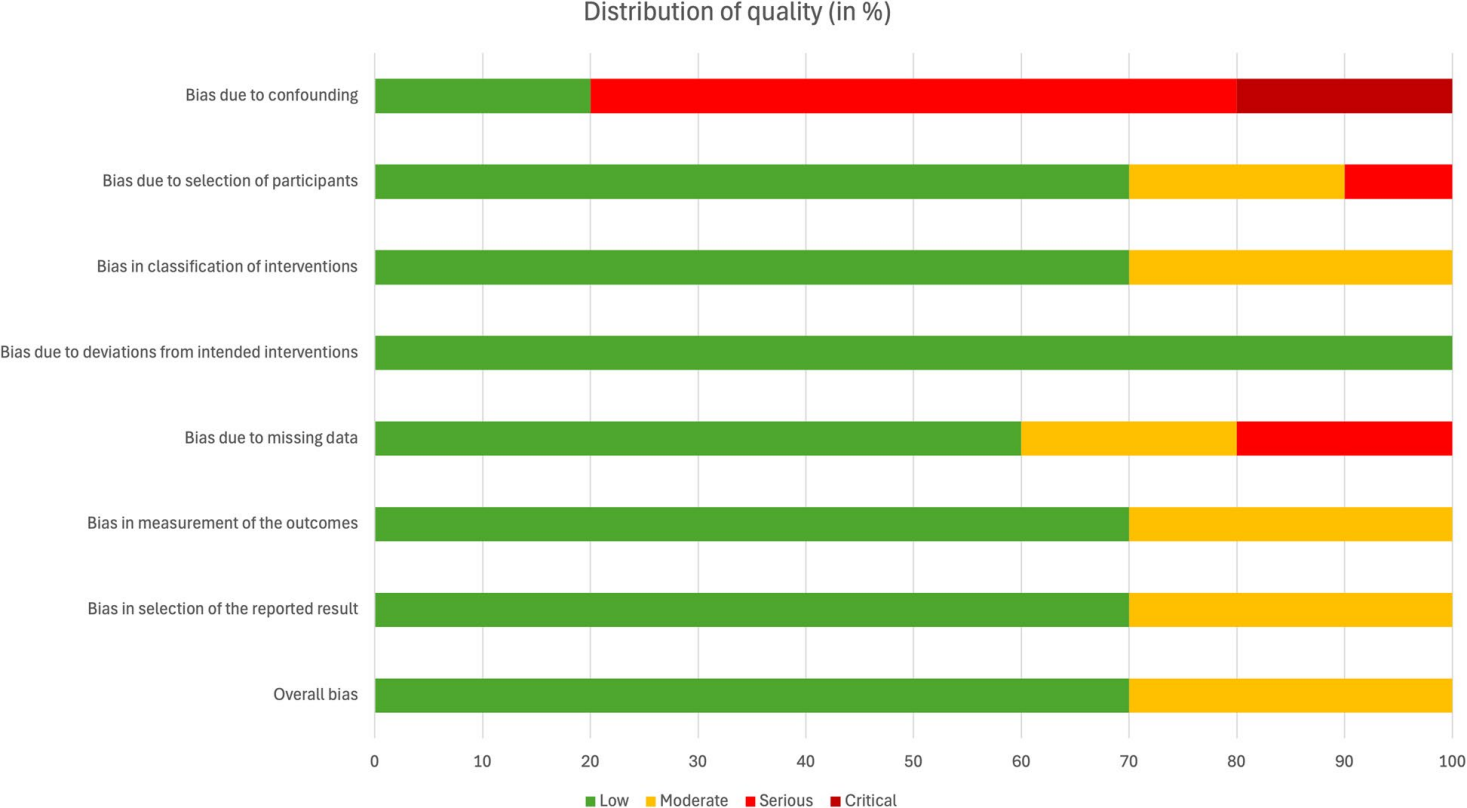
Distribution of quality assessments among the included studies (based on the ROBINS-I tool)

The selected studies were individually screened using the Risk Of Bias In Non-randomised Studies—of Interventions (ROBINS-I) tool [43]. Seven showed a low RoB, and three presented a moderate RoB (Table 3). The overall quality was good, and most concerns were due to confounding, the participant selection or missing data (Fig. 2).

**Table 3:**
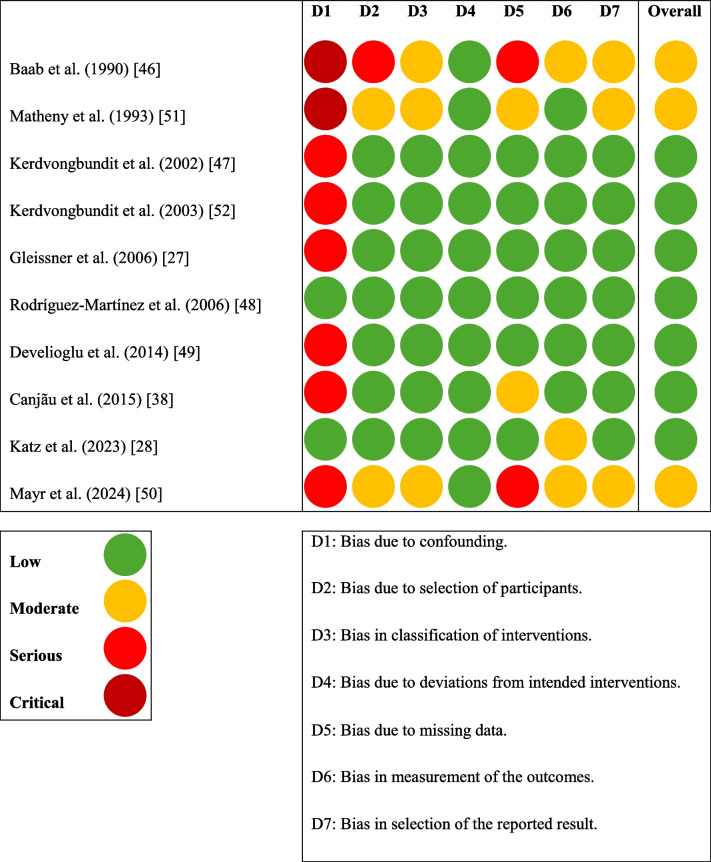
Individual bias of each included study based on the ROBINS-I tool

## Discussion

LDF is a commonly used tool in medicine, but dental use has increased for measuring hyperperfusion in the context of wound healing inflammation states. The aim of this review was to evaluate whether LDF is capable of detecting perfusion differences between sites of gingival inflammation compared to healthy controls and therefore a useful addition to clinical routine check-ups. To the best of our knowledge, two former narrative reviews by Kouadio et al. [26] and Orekhova et al.[29] investigated all kinds of intraoral indications of LDF. However, these studies did not directly compare LDF as a diagnostic tool for states of gingival inflammation and did not include recent studies systematically.

This review included studies using LDF devices from four different manufacturers, with distinct types of gingival probes which allows for meaningful within-study comparisons but prevents a meta-analysis due to methodological heterogeneity.

Several studies used splints or ligatures to stabilize the LDF probe [27, 38, 45–48, 50, 51], while other positioned the probe directly on the marginal gingiva with controlled pressure [49], or placed it on adjacent teeth to measure the papillae without applying pressure [28]. These methodological differences significantly affect the measurement outcome. For instance, Gleissner et al., demonstrated that perfusion values can differ markedly between handheld and splint-stabilized probes— potentially leading to artificial increases in blood flow readings due to motion or pressure artifacts [27].

Furthermore, the anatomical location of measurement (e.g., marginal gingiva vs. papilla) has a considerable influence on perfusion values [46], complicating comparisons across studies. Variability in probe positioning, type, and stabilization therefore presents a major challenge to reproducibility and data comparability in LDF-based research. Nonetheless, within-study comparisons still allow for the identification of general trends, such as increased perfusion in inflamed compared to healthy sites.

Gingivitis is known to be a reversible inflammation of the gingiva, that is accompanied by redness, swelling, and BOP [52], most studies showed increased blood flow in patients with gingival inflammation. Since LDF values are reported in AU and cannot be directly compared across different devices, only trends of increases and decreases can be evaluated between studies [26, 53].

Gingivitis can be classified as acute, experimental, or chronic [54]. While Gleissner et al. found significant differences in blood flow when comparing chronic gingivitis to healthy sites, values increased in experimental gingivitis, but with no significant difference [27]. Matheny et al. also found no significant difference between patients with experimental induced gingivitis compared to healthy patients, but they found a significant increase in the number of visible vessels [50]. These findings could be explained by former studies that found greater immune regulation and angiogenesis in chronic gingivitis than in experimental gingivitis [55, 56]. Nevertheless, the studies by Canjãu et al. [38] and Kerdvongbundit et al. [51], both of which included patients with naturally occurring gingivitis, showed that following periodontal treatment, blood flow values and clinical appearance normalized, with no significant differences remaining compared to healthy controls. 

Looking at periodontitis, which is accompanied by tissue destruction and attachment loss, Baab et al. showed in an early study that gingival blood flow in young patients with periodontitis recovered more rapidly after cooling than in the healthy controls [45]. This could be explained by a higher number of blood vessels in gingival connective tissue in periodontitis patients [57]. Kerdvongbundit et al. found no significant difference in blood flow values between patients with gingivitis and patients with periodontitis but significantly higher values in periodontitis sites than in healthy sites [46], which is in line with the studies by Mayr et al. and Develioglu et al., who also measured higher blood flow in patients with periodontitis [48, 49]. On the one hand, the shift from gingivitis to periodontitis can be fluent; on the other hand, overlying gingivitis can mask chronic attachment loss with acute marginal inflammation [58].

Rodríguez-Martínez et al. further divided patients into groups of healthy gingiva, isolated gingivitis (no periodontitis), isolated periodontitis (no gingivitis), and sites with both gingivitis and periodontitis to isolate the effect of periodontal attachment loss on blood flow changes [47]. They found significantly lower blood flow values not only in patients with isolated periodontitis but also in patients with a combination of both compared with healthy patients and patients with isolated gingivitis. Although periodontitis includes increased vessel density, it also causes vascular dysfunction and tissue damage, which could also explain lower blood flow in a chronic state [59]. Depending on the state of vessel degeneration and dysfunction, it can be difficult to identify the shift from chronic gingivitis to manifest periodontitis using perfusion measurements. BOP is the primary clinical parameter used to detect gingival and peri-implant inflammation, although it is known to have a relatively high rate of false positive or false negative results [8, 9]. Most studies included in this review used either the GI [27, 46–48, 50, 51] or PBI [28] to grade gingival inflammation. Only one study analyzed the correlation between LDF-based blood flow values and BOP reporting significantly higher flow at sites with positive BOP [28]. Three other studies found a significant association between higher GI scores and increased blood flow [27, 47, 48]. However, a major limitation across the current body of evidence is that most studies did not assess the relationship between LDF measurements and established clinical indices such as BOP, GI, or PBI. This lack of correlation with gold-standard diagnostics limits the interpretability and comparability of LDF findings and weakens the case for LDF as a standalone diagnostic tool in clinical settings.

Smoking and diabetes are known to alter gingival vessel architecture and lead to the degeneration of capillaries and chronic inflammation [60–63].

Concerning diabetes, Mayr et al. found no significant differences between medically healthy patients and patients with periodontitis affected by diabetes [49], which is similar to the findings of the study by Rodríguez-Martínez et al., who found that the patients with diabetes were more often affected by periodontitis, while gingival perfusion was similar between the medically healthy patients and the patients with diabetes [47]. Nevertheless, both studies included only a small number of patients with diabetes and the hemoglobin A1C (HbA1c) level was not documented to categorize the disease status. In contrast, Develioglu et al. only included patients with poorly controlled diabetes and an HbA1c level of > 8.5% and found higher blood flow in the patients with chronic periodontitis and diabetes compared to the chronic periodontitis alone or to the healthy controls [48].

Meekin et al. did not find any significant differences in gingival blood flow using LDF between non-smokers, light smokers, and heavy smokers [34]. In Katz et al.’s study, no significant differences were found in mean blood flow between the non-smokers and the smokers who had not smoked 2 h before the examination, but the study included a very small sample size of smokers [28]. In Mayr et al.’s study, no significant difference was found between the patients with periodontitis who smoked and the healthy patients, but a significant difference was found between healthy patients and non-smoking periodontitis patients [49]. This could suggest that smoking may mask the clinical signs of underlying periodontitis.

These findings suggest that both diabetes and smoking habits can affect perfusion values by increasing inflammation or causing vessel degeneration, which may either increase or decrease gingival blood flow. This should be considered when comparing perfusion measurements.

Important limitations of this review include the small sample sizes of most included studies, the use of different measurement locations, various LDF devices and probes, and the lack of regression analysis to control for potential confounding factors such as sex, age, biotype, smoking status, and diabetes.

LDF is a valuable tool for assessing relative changes in gingival blood flow, particularly in longitudinal measurements within the same patient—such as before and after gingivitis or periodontitis therapy—where device settings and measurement conditions can be kept constant. This intra-individual approach minimizes variability and allows for more meaningful comparisons. However, interpreting absolute perfusion values remains challenging. Perfusion is reported in arbitrary units (AU), which are influenced by technical factors such as device calibration, probe geometry, and measurement angle, limiting comparability across studies. Additionally, inter-individual variation and the absence of well-established reference values for healthy gingival tissue further complicate interpretation. Although some initial studies have attempted to define physiological baseline values [64–66], there is a clear need for standardized measurement protocols and normative data to enhance the diagnostic reliability of LDF in periodontal research.

Compared to other methods used to visualize and measure gingival perfusion, Doppler ultrasonography has also proven capable of detecting changes in gingival blood flow, as demonstrated in previous studies evaluating tissue perfusion after soft tissue augmentation at implant sites [19], as well as for monitoring peri-implant health [67, 68]. However, Doppler ultrasonography probes are generally larger than LDF probes. While this may reduce their sensitivity to motion artifacts, it also limits their applicability in the confined spaces around teeth or implants [27, 28, 67, 68]. Furthermore, their greater penetration depth makes them less suitable for assessing capillary-level microperfusion, particularly in the thin gingival tissues adjacent to teeth and implants [19, 28, 48, 67, 68].

Overall, LDF appears promising for research applications in detecting gingival perfusion changes, but its clinical applicability is currently limited. Traditional diagnostic methods such as periodontal probing and BOP, as endorsed by the 2017 American Academy of Periodontology and the European Federation of Periodontology (AAP/EFP) classification [69], continue to be the gold standard in daily clinical practice. Future studies are needed to further validate LDF in larger, controlled clinical settings before it can be considered a reliable tool for routine periodontal diagnostics.

## Conclusion

While LDF has been applied in various experimental settings to detect changes in gingival perfusion associated with inflammation, its clinical applicability remains limited due to considerable variability in probe types, measurement protocols, and stabilization methods across studies. The reviewed literature suggests that LDF may reflect increased blood flow in sites with chronic gingivitis and reduced perfusion following successful treatment. However, the transition from gingivitis to periodontitis appears less consistently detectable.

Future randomized studies using standardized LDF protocols— including probe stabilization (e.g., via splints), defined probe pressure, and controlled environmental conditions—are needed to assess reproducibility and diagnostic value. It is also essential to correlate perfusion data with clinical parameters such as BOP and gingival appearance, ideally through regression analysis. Furthermore, the potential differences in perfusion between natural teeth and dental implants remain largely unexplored and warrant investigation.

Overall, while LDF may offer additional insights in research contexts, it cannot currently replace traditional clinical diagnostic tools. Parameters such as probing depth and BOP, as emphasized in the 2017 AAP/EFP classification, remain the gold standard for daily clinical decision-making.

## Supplementary Information

Supplementary Material 1. Table 1 Full Search Strings by Database.

## Data Availability

The datasets used for the current study are available from the corresponding author on reasonable request.

## Abbreviations

LDF: Laser-Doppler flowmetry
PRISMA: Preferred Reporting Items for Systematic Reviews and Meta-Analyses
PROSPERO: International Prospective Register of Systematic Reviews
AU: Arbitrary Units
GI: Gingival Index
BOP: Bleeding on probing
PBI: Papilla Bleeding Index
ASA: American Society of Anesthesiologists
RoB: Risk of bias
ROBINS-I tool: “Risk Of Bias In Non-randomised Studies—of Interventions”
HbA1c: Hemoglobin A1C
AAP/EFP: American Academy of Periodontology and the European Federation of Periodontology
