# Supplementary material for: Laser doppler flowmetry as a diagnostic tool to detect gingival inflammation: a systematic review
Source: BMC Oral Health. 2025 Jul 31;25:1290. doi: 10.1186/s12903-025-06608-9 (PMC12315472; doi:10.1186/s12903-025-06608-9)
Supplement: Supplementary file 1 — Supplementary Material 1. Table 1 Full Search Strings by Database. [file 12903_2025_6608_MOESM1_ESM.docx]

**Supplementary Table 1** Full Search Strings by Database

| **Database** | **Search string used** |
| --- | --- |
| PubMed | ("laser Doppler flowmetry"[Title/Abstract] OR "laser Doppler"[Title/Abstract]) AND ("gingival"[Title/Abstract] OR "gingiva"[Title/Abstract]) AND ("perfusion"[Title/Abstract] OR "microcirculation"[Title/Abstract]) AND ("inflammation"[Title/Abstract] OR "gingivitis"[Title/Abstract] OR "peri-implant mucositis"[Title/Abstract] OR "peri-implantitis"[Title/Abstract] OR "periodontitis"[Title/Abstract]) |
| Embase | ('laser doppler flowmetry':ti,ab OR 'laser doppler':ti,ab)  AND  ('gingival':ti,ab OR 'gingiva':ti,ab)  AND  ('perfusion':ti,ab OR 'microcirculation':ti,ab)  AND  ('inflammation':ti,ab OR 'gingivitis':ti,ab OR 'peri-implant mucositis':ti,ab OR 'peri-implantitis':ti,ab OR 'periodontitis':ti,ab) |
| Cochrane Library | ("laser Doppler flowmetry" OR "laser Doppler")  AND  ("gingival" OR "gingiva")  AND  ("perfusion" OR "microcirculation")  AND  ("inflammation" OR "gingivitis" OR "peri-implant mucositis" OR "peri-implantitis" OR "periodontitis")  :ti,ab,kw |
| Scopus | TITLE-ABS-KEY("laser Doppler flowmetry" OR "laser Doppler")  AND TITLE-ABS-KEY("gingival" OR "gingiva") AND TITLE-ABS-KEY("perfusion" OR "microcirculation")  AND TITLE-ABS-KEY("inflammation" OR "gingivitis" OR "peri-implant mucositis" OR "peri-implantitis" OR "periodontitis") |

**Legend:**
[Title/Abstract] = term appears in the title or abstract (PubMed)
:ti,ab = title and abstract fields (Embase)
:ti,ab,kw = title, abstract, and keyword fields (Cochrane Library)

TITLE-ABS-KEY = term appears in the title, abstract, or keywords (Scopus)
